# Developing Anti-*Babesia bovis* Blood Stage Vaccines: A New Perspective Regarding Synthetic Vaccines

**DOI:** 10.3390/ijms24065219

**Published:** 2023-03-09

**Authors:** Laura Cuy-Chaparro, César Reyes, Eliana Vanessa Díaz-Guiot, Darwin Andrés Moreno-Pérez, Manuel Alfonso Patarroyo

**Affiliations:** 1 PhD Programme in Biotechnology, Sciences Faculty, Universidad Nacional de Colombia, Carrera 45#26-85, Bogotá DC 111321, Colombia; 2 Molecular Biology and Immunology Department, Fundación Instituto de Inmunología de Colombia (FIDIC), Carrera 50#26-20, Bogotá DC 111321, Colombia; 3 Structure Analysis Department, Fundación Instituto de Inmunología de Colombia (FIDIC), Carrera 50#26-20, Bogotá DC 111321, Colombia; 4 Animal Science Faculty, Universidad de Ciencias Aplicadas y Ambientales (U.D.C.A), Calle 222#55-37, Bogotá DC 111166, Colombia; 5 Microbiology Department, Faculty of Medicine, Universidad Nacional de Colombia, Carrera 45#26-85, Bogotá DC 111321, Colombia

**Keywords:** babesiosis, *Babesia bovis*, control strategy, vaccine, synthetic vaccine

## Abstract

Bovine babesiosis is caused by the Apicomplexa parasites from the genus *Babesia*. It is one of the most important tick-borne veterinary diseases worldwide; *Babesia bovis* being the species associated with the most severe clinical signs of the disease and causing the greatest economic losses. Many limitations related to chemoprophylaxis and the acaricides control of transmitting vectors have led to the adoption of live attenuated vaccine immunisation against *B. bovis* as an alternative control strategy. However, whilst this strategy has been effective, several drawbacks related to its production have prompted research into alternative methodologies for producing vaccines. Classical approaches for developing anti-*B. bovis* vaccines are thus discussed in this review and are compared to a recent functional approach to highlight the latter’s advantages when designing an effective synthetic vaccine targeting this parasite.

## 1. Introduction

The Apicomplexa phylum consists of a group of obligate intracellular pathogens that cause significant veterinary and human diseases, i.e., parasites from the genera *Babesia, Theileria, Plasmodium* and *Toxoplasma* [1]. Such parasites are characterised by having an apical complex consisting of the organelles (the rhoptries, the micronemes and the conoid organelles or the spherical bodies) which secrete molecules participating in the target cell adhesion/invasion and promote parasite development for producing the disease in a targeted host [2,3,4]. Such parasites are transmitted by the arthropod vectors, ticks being the main *Babesia* transmitting agent. More than 100 tick species have been reported to date, which are widely distributed throughout North America, South America, Africa, Asia and Australia [5,6,7]. *Rhipicephalus* (*Boophilus*) *microplus* infects a large variety of wild and domestic hosts (preferring cattle) and is a biological vector of the *Babesia bovis* species that causes bovine babesiosis in cows [8]. 

**Figure 1:**
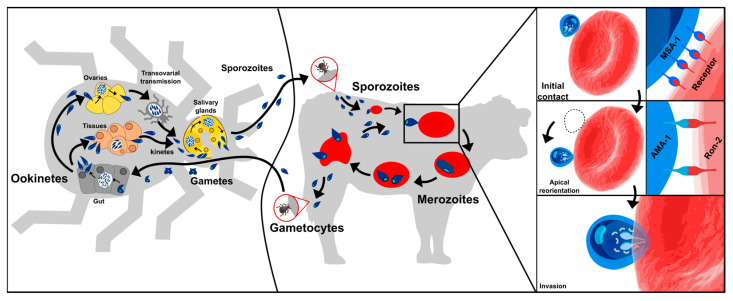
*B. bovis* life-cycle. Two hosts are involved; one is a vector (tick) in which the sexual part of its cycle takes place (left) and the other a vertebrate host (bovine) where asexual multiplication takes place (middle). Right panel shows proteins identified so far as participating in parasite initial contact and reorientation towards the apical pole.

*B. bovis* has a complex life-cycle requiring a vector where the parasite’s sexual reproduction occurs and a vertebrate host where asexual multiplication takes place (Figure 1) [9]. A vector’s bite inoculates *B. bovis* sporozoites (Spz) into a host’s bloodstream; these directly invade the erythrocytes by establishing the pathogen-cell molecular interactions. Once inside a cell, the Spz become freely located in the cytoplasm where they become the trophozoites, a few hours later [8,10]. This marks the beginning of the merogony phase, i.e., the parasite asexually reproduces by replicating its own nucleus inside its host’s cell. This is followed by the parasite inducing the cell segmentation (i.e., dividing by binary fission) to produce two infective merozoites (Mrz) which lyse the erythrocytes to be released into the host circulation and invade new erythrocytes. The invasion process proceeds continuously, giving rise to successive and asynchronous Mrz production; this leads to different parasitic stages being found in a host’s bloodstream [8,11] and causes clinical signs 7 to 35 days after parasite inoculation.

There has been controversy to date about the development of the pregametocytes within a vertebrate host’s bloodstream; there is no experimental evidence of this regarding *B. bovis* [8,11,12,13]. Nevertheless, when ticks feed on the blood forms, the male and the female gamete development begins in their gut lumen which fuse to form zygotes (known as the motile ookinetes) that invade the intestinal cells [13]. The motile ookinetes produce the kinetes (via meiotic division) which pass into the haemolymph and invade a vector’s peripheral tissues (including the salivary glands and the ovaries), thereby promoting transovarian and transstadial transmission. These two strategies are used by *B. bovis* for biological multiplication within the tick ovaries [14]. The kinetes become sporoblasts when they become dispersed to the salivary glands (i.e., asexual multiplication by sporogony), asynchronously producing 5000 to 10,000 Spz which are continuously released into a vertebrate host’s bloodstream when a tick is feeding [10]. Successful parasite invasion will depend on the use of its extracellular transmembrane proteins which interact with the host cell surface receptors, as in other Apicomplexa. However, the molecular mechanisms involved in the *B. bovis* life-cycle stages are yet to be fully understood.

*B. bovis* is considered to be the *Babesia* genus’ most virulent agent due to its production of severe clinical signs in infected cattle, such as haemolytic anaemia, haemoglobinuria, neurological syndrome and death [15]. Its vector is widespread in the tropical and the subtropical regions, leading to significant economic losses for the livestock industry worldwide [16,17]. The traditional bovine babesiosis control has mainly been based on the use of drugs and acaricides for the evading transmitting vectors [18,19]. However, many limitations, such as the appearance of drug resistance [20,21], the high treatment costs, the toxic environmental effects caused by using acaricides, the lack of knowledge about vector distribution and the socioeconomic and sanitary implications, have led to the live attenuated vaccine immunisation being adopted as an alternative control strategy. Nevertheless, the parasite invasion of the target cells cannot be completely controlled and, although other vaccine approaches focused on inhibiting parasite-cell interaction have been developed (such as recombinant proteins, viral vectors or synthetic peptides), this has highlighted the need for evaluating novel vaccine design methodologies.

A functional approach-based strategy has been evaluated due to the need for developing an effective anti-*B. bovis* vaccine; it involves identifying the conserved parasite molecule-derived fragments that play an important role in bovine erythrocyte adhesion-invasion [22]. Such an approach has led to the identification of protein conserved regions in other Apicomplexa that play a significant role in target cell binding and invasion, thereby enabling promising vaccine candidates for designing synthetic vaccines to be found. Previous findings suggest that this methodology could be considered as an appropriate strategy for designing a vaccine that can effectively control *B. bovis*-induced disease, having the advantages of safety, ease of production and efficiency. This review describes the current anti-*B. bovis* vaccine status and the potential value of the “functional approach” for designing an effective synthetic vaccine against this parasite.

## 2. Anti-Babesia Vaccines

**Figure 2:**
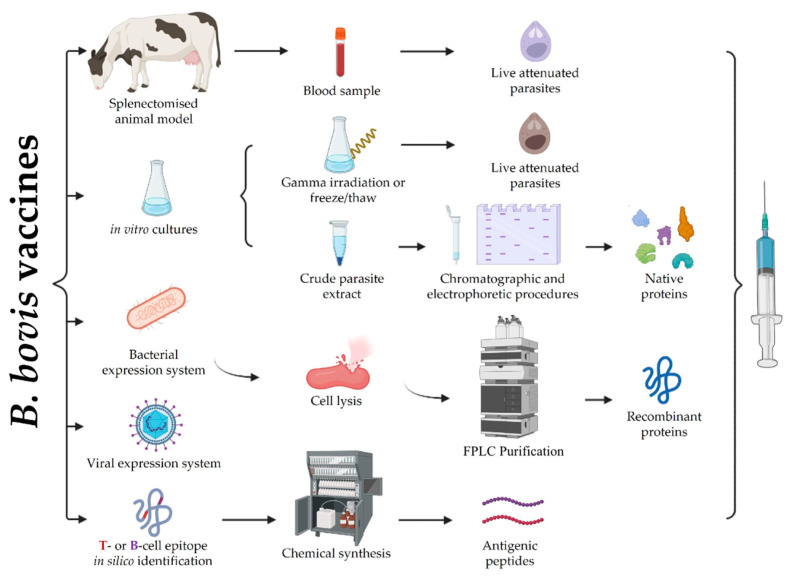
*B. bovis* vaccine design. The figure summarises the different anti-*B. bovis* vaccine types developed to date. Live attenuated vaccines are obtained by successive parasite passages in splenectomised cattle or in vitro cultured parasites that are subsequently attenuated by different methods. Protein-based vaccines have been obtained by size separation of crude parasite extracts, recombinant expression in *E. coli* and subsequent lysis and purification by affinity chromatography, or viral vectors engineered to express the protein if interest in vivo. Peptide-based vaccines are focused on the in silico identification of short regions containing B- or T-cell epitopes and subsequent chemical synthesis. Figure created with BioRender.com.

Resistance to drugs targeting *B. bovis* or its transmitting vector has made vaccination against this parasite the main infection control method [23]. The main vaccines developed and introduced in several countries for many years to date have involved using live attenuated parasites obtained from passages in splenectomised cattle (Figure 2) [24,25]. Other types of vaccine have also been studied, such as attenuated parasite-based vaccines obtained from in vitro cultures, or recombinant proteins/viral vectors or peptide-based vaccines targeting the *B. bovis* blood stage; however, these types of vaccines have only been used in scientific studies to date [23,26,27]. Even though these types of vaccines have induced considerable levels of protection (especially in calves under a year old), they have not been completely effective and the production-related difficulties have limited their commercialisation.

### 2.1. Live Attenuated Parasite Vaccines

This type of vaccine emerged from the observations made in 1899 concerning the fact that long-lasting immunity against new infection was induced in animals that had recovered from natural *Babesia* infection. Furthermore, subsequent evidence showed that blood from infection-recovered animals did not produce a severe form of disease in recipient animals [28]. The live attenuated parasite vaccine systems have involved using low virulence *B. bovis* strains that have been attenuated through successive passages in splenectomised cattle or by using in vitro cultures that were kept frozen for several days or by deep freezing for long periods in liquid nitrogen (Figure 2) [29]. *B. bovis* B, C and D strains attenuated using the aforementioned procedures (isolated from natural infection during babesiosis outbreaks in various regions of Australia) were evaluated around 1957, and demonstrated good efficacy and safety results [29]. However, it was reported that no more than 30 passages should be carried out in cattle since a greater amount considerably affected effectiveness in immunised cattle [30]. A study that involved inoculating 189 cattle (distributed into 5 trials) with 5 independent vaccine strains found that the animals developed differing degrees of protection against the experimental challenge; the T strain provided high protection, the Kr strain induced moderate protection whilst the L, the B and a mixture of the Ka and the B strains were poorly protective, the latter because they were most likely infected with antigenically distinct *B. bovis* strains [31].

Developing a *B. bovis* in vitro culture has been another alternative used to facilitate attenuated live vaccine design and to evaluate its usefulness for inducing an immunogenic response during the experimental infection. For example, one study has shown that inoculating the *B. bovis* 429 strain (attenuated and adapted to grow in equine serum) into 4 splenectomised calves reduced the clinical signs of infection when the animals were challenged with the virulent *B. bovis* KB strain 44 and 78 days after the vaccination [32]. Another study demonstrated no clinical disease in animals immunised with the BOR clone (derived from a radiation-attenuated, *B. bovis* KBb strain in vitro culture) when they were challenged with a homologous strain, this being consistent with strain-specific immunity [33].

Such results suggest that an in vitro culture represents an alternative for obtaining antigens similar to those in in vivo culture which retain their functional properties [34,35]. The use of in vitro culture has advantages over the in vivo culture system given that it reduces animal usage by producing the attenuated strains in vitro; it also enables vaccines to be manufactured in more controlled and standardised conditions and implies less risk of pathogen co-transmission (due to measures such as gamma irradiation of the serum used for the parasite culture) [26,36]. Live frozen vaccine production facilitates quality and safety tests to be undertaken before the product has been delivered and inoculated, has a long shelf-life (which can be extended for up to 18 months stored in liquid nitrogen) and involves lower production costs compared to those for the refrigerated vaccines [37].

Immunisation with live attenuated *B. bovis* microorganisms targeting this species has been used in Australia, Brazil, Israel, Mexico, Colombia, Argentina, South Africa and Uruguay. In addition to the aforementioned advantages, it must be stressed that this type of vaccine involves significant limitations related to its production and administration. For example, production of this type of vaccine requires fresh bovine erythrocytes and serum from specific donors requiring very strict production/maintenance conditions to ensure that they are pathogen-free (which can be difficult when the biological product is made in tick-endemic regions) and guaranteeing parasite viability from the beginning of preparation until the final product delivery. In vitro cultured parasite immunogenicity may be lost after long-term maintenance in such conditions; access to suitable laboratory equipment and the availability of trained personnel is necessary, given the complexity of maintaining a *B. bovis* culture for large-scale vaccine production [23,27]. Regarding administration, there are still difficulties related to standardising the dose required for the vaccination. There is also the potential risk of tick transmission of the vaccine strain that could recombine with the field strains, potentially leading to new virulent variants or virulent reversion [23,26]. The ideal moment for immunisation must be taken into account; administration to weaning age calves being preferred because adult cattle can be highly susceptible to vaccine components inducing serious reactions, such as abortion [38]. Although several changes have been introduced to improve live attenuated vaccine quality in terms of the different production stages, the many disadvantages of such vaccines have prompted research into alternative methodologies facilitating production, offering better safety ranges and ensuring more homogeneous efficacy against *B. bovis* infection [37].

### 2.2. Protein-Based Vaccines

The main molecules activating an immune response are the parasite proteins expressed during the blood stages; there has thus been major interest in studying complete recombinant molecules or their fragments which can activate the cellular and the humoral immune responses controlling infection [39,40]. This approach has been useful, given that the parasite proteins/fragments can be obtained individually, involving low production costs using different expression systems (*Escherichia coli* being the most used), along with the relative ease of standardising the production and storage and a greater degree of safety compared to that for live vaccines [41].

Affinity chromatography purification and identification of the antigens obtained from the *B. bovis* crude extracts formed the first approach involving this type of vaccine. This assay involved the sequential vaccination/challenge experiments in adult cattle using crude parasite extracts systematically fractionated by chromatographic and electrophoretic procedures (Figure 2); fractions eliciting a protective response were used for purifying the antigens by pull-down assays (i.e., 11C5 and 12D3), using the monoclonal antibodies (Ab) developed against these parasite fractions. Interestingly, immunising recombinant 115C and 12D3 antigens in combination induced an immune response which was able to decrease parasitaemia levels in cattle exposed to the homologous *B. bovis*, being similar to that obtained using the commercially-available live attenuated vaccine [42]. However, protection levels became reduced when an extremely virulent heterologous *B. bovis* strain (W strain) was used to challenge the individuals previously immunised with 11C5 and 12D3 antigens (combined or individually) due to the calves developing parasitaemia. As such, these results highlight the notion that these fragments induce strain-specific immunity [43].

The rhoptry associated protein 1 (RAP-1) has been confirmed as an antigen containing the immunodominant and conserved T-cell epitopes in its N-terminal region which can induce a response from the IFN-γ-producing CD4^+^ T-cells and increase IgG2 synthesis, thereby conferring partial protection in animals challenged with the parasite [44,45,46]. Nevertheless, such a response did not guarantee protective immunity against challenge with the virulent *B. bovis* strain, nor did it change the infection’s clinical course [47]. Weaker protection has also been observed when comparing the protective immunity attained between groups of cattle vaccinated with the live attenuated parasites and the recombinant RAP-1, since 100% of the animals immunised with this protein developed the disease and 50% of them required pharmacological treatment compared to 80–100% effectiveness using live calf-derived parasites [38].

Merozoite surface antigen-1 (MSA-1) has also been of interest given that it is mainly exposed to the immune system during natural infection. However, despite MSA-1 being able to induce neutralising Ab production in vitro [48], it did not protect calves immunised with three doses of recombinant saponin-emulsified MSA-1 after they were challenged with the *B. bovis* T2Bo strain [49]. Such findings could be explained by limitations such as the brief period of exposure to Mrz in serum, thereby limiting accessibility to neutralising epitopes [50], difficulties related to producing protective Abs (due to high MSA-1 variability inducing strain-specific responses and thus the lack of cross-protection), or the presence of other proteins from the *B. bovis* variable merozoite surface antigen (VMSA) family functionally replacing MSA-1 proteins.

The apical membrane antigen-1 (AMA-1) is a very interesting molecule as its evaluation in *Plasmodium* has shown that it plays an indispensable role in the host cell invasion. It has been found that the Ab production induced by the *B. bovis* recombinant protein encoded by the conserved central region (*Bb*AMA-1 ectodomain I (DI)and II(DII)) significantly inhibited Mrz growth as well as the efficiency of in vitro Mrz invasion of the bovine erythrocytes by 70% after 6 h, suggesting that this region is a good candidate for inclusion in an anti-*B. bovis* vaccine [51]. Such findings are consistent with a recent study which showed that cattle vaccinated with *Bb*AMA-1-DI/DII developed the humoral and the cellular responses which could be associated with protection [52].

One study has evaluated whether a mixture of such antigens is enough to increase the protection levels in immunised cattle. Alvarez et al., immunised cattle with a mixture of MSA-1, MSA-2c and 12D3 recombinant proteins emulsified with the Montanide adjuvant, administered in two doses. Despite animals becoming seroconverted from day 7 post-challenge onwards, they developed clinical symptoms after challenge with the *B. bovis* Yucatan strain [53]; this supports the belief that there is not enough evidence regarding the usefulness of these molecules as anti-*B. bovis* vaccine components.

Several studies have been aimed at establishing the relationship between vaccination and the underlying immunological mechanism clearing infection, since native/recombinant protein-based vaccines have not been able to completely prevent cell invasion/infection. One study reported that calves under the age of six months were relatively more resistant than adult cattle to developing severe forms of the disease after the initial *B. bovis* infection. Such age-related resistance is due to the maternal Ab protective effects and a spleen-dependent innate immune response inducing overproduction of mediators, such as IFN-γ, TNF-α, nitric oxide (NO) and macrophage activation. These activated macrophages secrete IL-12 and IL-18 that promote microorganism death by phagocytosis and the differentiation of natural killer (NK) cells for controlling the disease [54]. It has also been suggested that protection against *B. bovis* requires both an innate and an adaptive response through the CD4^+^ Th1 lymphocyte and the B-cell activation producing neutralising Ab targeting extracellular Mrz and the surface proteins [55]. Torina et al., found that an adaptive immune response depended on parasite antigen presentation to the CD4^+^ T-cells by professional antigen-presenting cells in immunised or persistently infected animals. Thus, infection control is probably mediated by activated splenic macrophages destroying the infected erythrocytes and by IFN-γ production potentiating an opsonising IgG2 Ab response targeting the extracellular Mrz surface antigens [56].

The above points support the notion that any vaccine formulation (antigens, adjuvants and delivery systems) should target enhancing interaction with antigen presenting cells (either dendritic cells or macrophages). Despite the above, protein-based recombinant vaccines have not been able to induce fully efficient immune responses and have not provided lasting protection. This may have been due to poorly folded or incorrectly processed membrane antigens giving rise to structural differences between native and recombinant proteins, thereby stimulating Ab responses having different specificities [57]. Furthermore, the antigen is not the only requirement responsible for inducing an immune response, since recombinant vaccine formulation usually involves using adjuvants (aimed at recruiting an innate immune response) and/or delivery systems (for increasing bioavailability into or throughout the body and for the stability of the whole formulation), which are used to refine and enhance their immunological behaviour. Further in-depth knowledge about the *B. bovis* biology and understanding the host immunity mechanisms will enable the efficient design of vaccines for controlling babesiosis.

### 2.3. Recombinant Viral Vector Vaccines

Recombinant viral vector vaccines seek to enhance humoral and cellular immune responses by using non-pathogenic viral vectors as expression platforms [58]. For example, the highly attenuated modified vaccinia virus Ankara (MVA) is a non-replicative vector used to express single or multiple immunodominant antigens in vivo (Figure 2). Once MVA has infected host cells, recombinant proteins are expressed and exhibited to the immunological system to trigger responses, mainly Th1-type [59].

The following factors have made recombinant viral vectors promising tools for designing vaccines: their strong immunogenicity is provided by poxvirus vectors; there is high-level recombinant protein production when using the MVA system in a eukaryote system; the production costs are relatively low; the stability of the system; there are high safety levels for both laboratory personnel and those receiving the vaccine and there is ease of administration [58,60]. A study evaluating the humoral and the cellular immune responses in mice immunised with a chimeric multiantigen consisting of *B. bovis* MSA-2c, RAP-1 and the small heat shock protein 20 (HSP20) immunodominant regions expressed by a recombinant MVA vector has shown that a heterologous vaccination scheme involving a recombinant virus booster induced high specific IgG Ab titres and the activation of the CD4^+^ and the CD8^+^ T-cells producing TNF-α and IFN-γ, thereby boosting an immunological response against *B. bovis* [61]. Another study involved immunising a group of steers twice at 42-day intervals with a live attenuated R1A vaccine and another with a primary subunit vaccine containing MSA-2c, RAP-1 and HSP20 *B. bovis* protein B- and T-epitopes, followed by a booster dose 42 days later with the Ankara vector expressing the same antigens. The animals which received the live R1A vaccine did not develop clinical symptoms of the disease in contrast to those receiving the subunit vaccine which showed a remarkable and rapid onset of clinical signs after challenge with virulent *B. bovis* S2P [62]. Both the groups had comparable IgG titres having an antigen-specific Th1 cell response at the end of the trial. Interestingly, the live attenuated R1A vaccinated group developed the neutralising Ab in vitro which inhibited bovine erythrocyte invasion by 79.4% compared to 44.4% for the subunit vaccine group. Future studies should concentrate on improving the immune response stimulation using this system.

### 2.4. Antigenic Peptide-Based Vaccines

The chemical synthesis of short sequences containing the T- and the B-cell recognition sites has been established as a strategy for vaccine development (Figure 2) as only these molecule’s fragments can induce an immune response [63]. This approach has been studied with proteins participating in the parasite-erythrocyte interaction and is based on evidence related to their antigenic role during natural infection (described in a previous section).

Several peptides containing RAP-1, MSA-2c and AMA-1 B-cell epitopes were able to trigger neutralising Ab in cattle, inhibiting in vitro invasion by 34.7% on average (using RAP-1 P1 and P4), 6.28% (with AMA-1 P2) and 10.34% (MSA-2c P3 and P4) when used individually and 52.37% when evaluated as a pool. Interestingly, MSA2c P3 and AMA-1 P2 induced gamma interferon (IFN-γ) production in PBMCs from the vaccinated cattle after one year, thus providing a long-lasting Th1 immune response [64]. It has been shown that a recombinant epitope derived from the *Bb*AMA-I-DI surface-exposed α-helix (specifically in the PAN motif) was able to elicit Ab in rabbits which significantly inhibited the in vitro parasite invasion by 60% within 4 h of incubation and Mrz growth by 50–70% on days 3 and 4 of culture [65]. Characterising the *B. bovis* rhoptry neck protein 2 (RON2) ascertained its immunogenic capacity; several conserved synthetic peptides containing the T- and the B-cell epitopes have thus been used in immunisation assays in cattle. The results showed that the individual and the combined peptides were able to induce Ab after four immunisations when emulsified in Montanide ISA 71 adjuvant; they could inhibit parasite invasion of erythrocytes in vitro, with better results being obtained with the peptide combination (42% invasion inhibition) [66].

The above studies highlighted short fragment usefulness as anti-*B. bovis* vaccine components and has aroused great interest in the field of veterinary vaccinology due to advantages regarding greater stability, less complexity by using small regions instead of the complete antigen, ease of production and administration, inducing a specific immune response and safer and more effective products thereby being obtained for the livestock industry [67,68]. However, the appropriate parameters should continue being studied, based on the absence of a reliable correlation of protection with peptide use, in order to improve identifying potentially useful peptides for vaccine development, and having efficiency close to or better than that obtained by using live attenuated vaccines.

## 3. Functional Approach

Knowledge about the biology of the parasites belonging to the *Theileria, Plasmodium, Toxoplasma* and *Babesia* genera has advanced with different intensity, *Plasmodium* being one of the most studied due to its significant impact on human health worldwide [69]. *Plasmodium falciparum* and *Plasmodium vivax* are the most important species causing the highest mortality and morbidity rates worldwide, whilst *Plasmodium ovale*, *Plasmodium malariae* and *Plasmodium knowlesi* have the lowest prevalence [70]. Different strategies have also been studied for developing a vaccine as the main disease control measure. However, a new and robust functional approach has been proposed during the last few years; it has used *P. falciparum* as a malaria disease model. Rather than identifying variable regions, it is based on selecting critical protein-derived conserved regions related to parasite adhesion [71]. These regions were initially identified in *P. falciparum* proteins by screening the erythrocyte binding properties of 20-residue-long, non-overlapping peptides spanning the entire molecules. A sensitive and specific radio-iodination-based binding assay was used. Interestingly, conserved high activity binding peptides (cHABPs) to the erythrocytes were identified; these had to be modified to fit inside the major histocompatibility complex (MHC) II, thereby triggering a strong protection-inducing response. This methodology has enabled the identification of 100 Mrz protein-derived cHABPs which induced high Ab titres and protection when challenged in the *Aotus* monkey experimental model. They also had strong capability for inhibiting red blood cell invasion in vitro. Such characteristics support peptide suitability for inclusion when designing an efficient vaccine [71].

**Figure 3:**
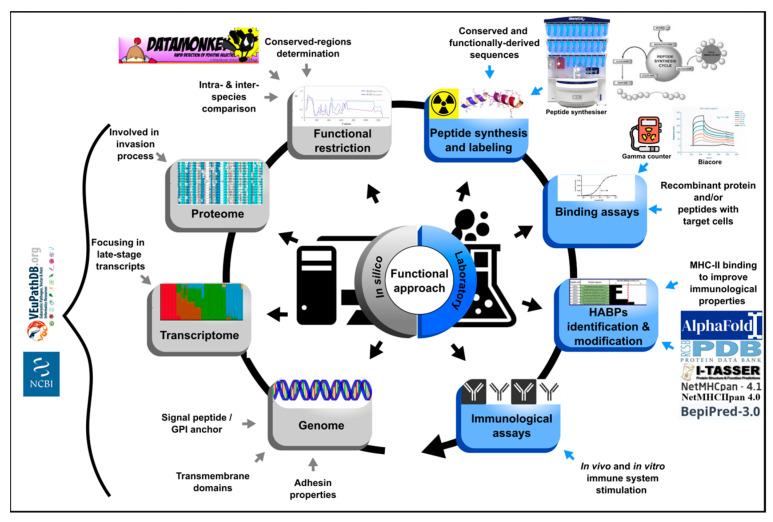
Functional approach strategy. Flow diagram describing the steps for prioritising in silico selection and laboratory experiments for identifying ideal vaccine component peptides. The strategy involves identifying the entire gene repertoire (using genome, transcriptome and proteome data mining) and their encoding sequence analysis by bioinformatics tools based on in silico characteristics and their intra (from 5 genomes)- and inter-species (phylogenetically related) conservation level [73]. Peptides encoded by regions under functional constraint are then chemically synthesised and target cell binding activity must be tested by radio-iodination-based peptide-cell interaction and competition assays [22]. Identified HABPs must be evaluated in in vitro culture and then modified to fit inside MHC-II to evaluate their role as vaccine component in in vivo tests in the pertinent experimental model. The figure shows the required resources for carrying out the functional approach.

This approach has been used and adapted for identifying minimal regions involved in *P. vivax* binding to the reticulocytes (Figure 3) [72]. Omic study results have been compiled as a strategy for filtering those regions expressed during the parasite’s life-cycle, that have the potential of participating in target cell entry. Bioinformatics tools are used to prioritise the candidate selection based on their in silico characteristics (such as the signal peptides, the membrane anchor sequences, the functional domains, the stage-specific expression profiles and the specific location pattern) and intra- and inter-species conservation level, the latter determined by natural selection analysis [73]. The protein’s target cell binding role is then evaluated using only 20-residue-long, non-overlapping peptides derived from the regions under functional constraint by the protein-cell interaction assays, like those performed for *P. falciparum*. This has enabled finding *P. vivax* cHABPs to target cells; their role in cell invasion must be confirmed in vitro and in vivo assays using modified peptides in the latter to improve their antigenicity. Unlike results obtained by the classical approach to designing vaccines based on proteins and peptides, in vitro results obtained by the functional approach are expected to be consistent with those in vivo, as confirmed for *P. falciparum*.

Such methodology has also been used with *B. bovis* and has returned promising results since several cHABPs binding to MSA-1- and AMA-1-derived bovine erythrocytes have been identified. The in silico approach was developed using the information regarding 5 genomes from different geographical isolates and the *Babesia orientalis* genome, a phylogenetically related species. Thus, several MSA-1 and AMA-1 peptide-coding gene regions were predicted as conserved and under negative-selective pressure using the online bioinformatic tools; functional activity was confirmed by peptide-cell interaction assays, as described for *Plasmodium*. MSA-1-derived peptides specifically bound to a sialoglycoprotein and 42422 and 42426 had a helical structure and conserved motifs in all strains/isolates [74]. Only 42437 and 42438 AMA-1-derived peptides bound to a chymotrypsin and neuraminidase-treatment sensitive receptors; they were exposed on protein surface (as confirmed by 3D structural prediction) and were composed of B- and T-cell epitopes [75]. These finding highlight the fact that functional subunits used by *B. bovis* for establishing receptor-ligand interactions can be predicted by the natural selection analysis. Previous studies in *P. falciparum* have found that functionally-relevant and conserved HABPs tend to be poorly immunogenic and protective and thus, have to be modified to better stimulate a strong immune response; it remains to be seen whether the naturally occurring B- and T-cell epitopes found so far in *Babesia* cHABPs will be immunogenic and protective when tested, or if they have to be modified for a better presentation in the MHC class II molecule. The bovine MHC-II 3D structure must be ascertained or an in silico bioinformatics modelling programme developed to make modifications to cHABPs and thus evaluate their usefulness in anti-*B. bovis* vaccine development.

## 4. Discussion

Veterinary vaccine development has been considered an effective and cost-effective strategy for infectious disease control (i.e., babesiosis); classical approaches based on immunising live attenuated parasites or parasite proteins/fragments have been the most studied to date [23]. However, even though live attenuated and recombinant vaccines have provided a percentage of protection in immunised animals, their limitations have highlighted the need for adopting alternatives in order to provide safer and more efficient vaccines [76].

The publication of pathogen genomes, coupled with bioinformatics strategies, has led to new immunogens being identified more easily, genetic variability being analysed and conserved protein functional regions being characterised (which are important for target cell invasion and are thus of interest for including in vaccine design) [77,78,79]. In silico analysis has revealed significant properties related to antigen expression, has facilitated T- and B-cell epitope mapping and predicting MHC complex (class I and/or II) molecule binding capacity for inducing a potent, long-lasting immune response.

Along with inducing a specific immune response, synthetic vaccines can facilitate the inclusion of multiple epitopes to improve antigenic diversity coverage during the infectious agents’ life-cycle stages. The use of oligomerisation systems enables many peptides to be coupled to a central matrix for increasing the peptide immunogenicity. Peptides having short amino acid sequences containing T- and B-epitopes have thus been designed and chemically synthesised to stimulate the cellular and the humoral immune responses [80]. Compared to the first-generation vaccines, the peptide-based vaccines have better stability, can be accurately physically-chemically characterised and involve lower risks of inducing adverse effects when purified, i.e., allergic reactions. Peptide-based vaccines can be reproduced easily, their production system is simple, fast and relatively cheap and chemical synthesis avoids contamination with derivatives from other biological agents [68].

It has been observed that the individual peptides’ immunological responses are usually not good, even though this type of vaccine’s advantages are self-evident. Therefore, the individual peptides have to be coupled to larger proteins or administered in combination with adjuvants to facilitate antigen presentation and protect them from enzyme degradation; the choice of adjuvant is therefore fundamental for a vaccine’s success [80,81,82]. Such an approach has been tested in both humans and animals, with promising results; however, further research is needed on new antigens and other administration technologies protecting active ingredients and promoting the suitable/desired immune responses [83].

*B. bovis* biology and the molecular mechanisms involved in its invasion are not fully understood. A few characterisation studies to date have described the *B. bovis* proteins and have only suggested a role during the initial contact and the tight-junction formation with the bovine erythrocytes for MSA-1 [49,84], AMA-1 [51], RON2 [66] and TRAP [51]. The cHABPs binding to the bovine erythrocytes containing T- and B-cell epitopes have been identified recently in *B. bovis* AMA-1 and MSA-1; they are being studied to confirm their usefulness as potential components for inclusion in a vaccine targeting the parasite [74,75]. However, the functional approach has not been adopted for studying other molecules. Identifying the above molecules’ function with respect to parasite invasion of bovine erythrocytes will facilitate the discovery of suitable peptides for studying their usefulness as anti-*B. bovis* vaccine components, along with novel, invasion-related, protein-derived peptides.

## 5. Conclusions

Anti-*B. bovis* vaccine development has been focused on live-attenuated parasites, recombinant proteins and antigenic peptides. However, such traditional strategies have highlighted the variability of these approaches, along with the many limitations related to their success. This has emphasised the need for adopting safe alternative vaccine design approaches, capable of inducing a long-lasting protection-inducing immune response. A functional approach is thus worth considering, taking into account the implicit advantages of safety, efficiency, ease of maintenance and production. Future studies for discovering suitable, invasion-related, protein-derived cHABPs will provide essential information for designing an effective vaccine against *B. bovis*.

## Data Availability

Not applicable.
